# Evaluation of uric acid disorders and associated factors in essential hypertensive patients at Wolkite University specialized hospital, Southern Ethiopia

**DOI:** 10.1371/journal.pone.0256557

**Published:** 2021-09-10

**Authors:** Abebe Timerga, Kassahun Haile

**Affiliations:** 1 Department of Biomedical Science, College of Medicine and Health Sciences, Wolkite University, Wolkite, Southern Ethiopia; 2 Department of Medical Laboratory Science, College of Medicine and Health Sciences, Wolkite University, Wolkite, Southern Ethiopia; Sao Paulo State University (UNESP), BRAZIL

## Abstract

**Background:**

Essential hypertension is a condition characterized by a rise in blood pressure of undetermined cause, includes 90% of all hypertensive cases and is a highly important public health challenge with major modifiable cause of morbidity and mortality. Uric acids disorders in particular hyperuricemia are significant problems in essential hypertensive patients and can cause substantial morbidity and mortality. Determination of uric acid disorders may play a major role in the management and early aversion of complications in hypertensive patient. Therefore, this study aimed to determine uric acid disorders and associated factors among essential hypertensive adults in the outpatient department at Wolkite University specialized Hospital, Southern Ethiopia from November 1 to February 30, 2021.

**Methods and materials:**

An institional based cross sectional study was conducted on 270 essential hypertensive adults on follow-up in outpatient department from November 1 to February 30, 2021. Structured questionnaires through face to face interviews and participants’ medical records were used to collect information on determinants related with uric acid disorders. The blood specimen was collected and level of serum uric acid, blood sugar and lipid profile was measured using standard principles and procedures with an ABX Pentra 400 automated chemistry analyzer. Bivariate and multivariate logistic regression analyses were done to identify factors associated with hyperuricemia. The p-value was set at <0.05 with a 95% confidence interval of the adjusted odds ratio.

**Results:**

A total of 270 adult essential hypertensive patients were participated in the study, among those 196(27.4%) of study participants were hyperuricemic with 95%CI (21.9, 33.3). Being alcoholic [(AOR: 15.68, 95% CI: (5.93, 21.41)], taking antihypertensive medication [(AOR: 11.56, 95% CI: (3.94, 23.80)], BMI > = 30 [(AOR: 4.89, 95% CI: (1.46, 25.5)] and being centrally obese [(AOR: 6.87, 95% CI: (2.53, 18.63)] were factors significantly associated with hyperuricemia.

**Conclusion:**

In this study, the high burden of hyperuricemia (27.4%) was observed in essential hypertensive patients with follow-up in outpatient department. Taking alcohol and antihypertensive medication, being overweight and centrally obese were identified factors of uric acid disorders. The finding of this study should be taken into consideration to implement preventive interventions on identified predictors in hypertensive patients. Taking fruit and vegetable, and promoting physical exercise and determinations of serum uric acid level in adult essential hypertensive patients was recommended to minimize the emergence of hyperuricemia.

## Introduction

Essential hypertension is a rise in blood pressure with overall prevalence of 26.4% with undetermined cause that includes 90% of all hypertensive cases and is a highly important public health challenge in its burden of morbidity and mortality because of its complications, including cardiovascular, cerebrovascular, and renal diseases. In (≈90%) of individuals, its etiology cannot be determined; therefore, the essential hypertension term is employed [1]. It is (80–95%) more common than secondary hypertension (5–20%) which is due to other aetiologies that raises blood pressure [2].

Uric acid (UA) is the end product of purine metabolism in humans, and nearly 70% of UA are eliminated by the renal system, while the rest is eliminated by the intestines [1,3]. Uric acid acts as an antioxidant (> 50% of the antioxidant capacity of the blood) by exerting a strong reducing effect together with bilirubin and ascorbic acid vitamin C) during the early stage [4]. When uric acid levels are too low and in its latter stage, the reducing effect also decreases. As a result, reactive oxygen products cannot be scavenged and neutralized which finally leads to the development of oxidative stress [5,6].

Several studies have been documented that the elevated serum uric acid (SUA) level is associated with the development essential hypertension itself or its complication, cardiovascular disease and the risk factors of metabolic syndrome [7,8]. Uric acid has several reported effects by which it may play a pathogenic role in development of hypertension [6,9]. Elevation of SUA (hyperuricemia) most likely caused by westernized lifestyle and increases with age, urbanization and with the presence of metabolic syndromes [10,11].

In human physiological solubility of uric acid occurs at 6.4 mg/dl, but the presence of uric acid-binding proteins increases this solubility to 7.0 mg/dl before reaching supersaturated state [12,13]. For this reason, hyperuricemia occurs when the serum level of uric acid exceeds 7.0 mg/dl, at which point it starts to crystalize within the body cells. However, an increament of serum uric acid level is considered to prohibit the increased risk of disease associated with adult lifestyle habits even when the serum level of uric acid is ≤7.0 mg/dl [14,15]. Hyperuricemia is also a significant predictor for coronary artery disease [16,17]. Compared to men, women require critical attention due to the increased disease risk even at lower serum uric acid levels [18]. Humans’ body cells are liable to develop higher serum UA than other mammalians because of the absence of urate oxidase that converts UA to allantoin during the course of evolution [19,20].

In hypertensive patients, monosodium urate crystals are deposited and easily precipitated on the vascular wall of blood vessels, due to mechanical stimuli caused by blood pressure that affect blood coagulation, which likely leads to arteriosclerosis and other related cardiovascular complications of hypertension with poor prognosis [21,22].

Even though, knowing the status of serum uric acid level and associated factors in patients with essential hypertension is crucial, the problems were not well studied in our setup and scanty with conflicting data are available. So, conducting this study is crucial to improve early diagnosis, prevention of complications and enhance quality of life of hypertensive patients. Therefore, this study was conducted to assess the prevalence of uric acid disorders and associated risk factors in essential hypertensive patients in outpatient department at Wolkite University specialized hospital (WKUSH).

## Materials and methods

### Study design, area and period

The study was conducted at Wolkite University specialized hospital from November 1 to February 30, 2021. The study was conducted on essential hypertensive patients in outpatient department attending their follow-up at WKUSH. Facility-based cross-sectional study design was conducted to carry out the project.

### Sample size and sampling techniques

A single population proportion formula was used to calculate the required sample size by considering the following assumptions: The prevalence of hyperuricemic among essential hypertensive patients = 31.5% [23], 95% confidence level, 5% degree of precision, and non-response rate of 10% which gives 342. Finally, population correction formula was employed since the population is less than 10,000 (i.e. 868) and the final sample size for the study was 270. All consecutively identified essential hypertensive patients who fulfilled the inclusion criteria were enrolled in the study.

The source population of this study was all patients attending outpatients department of WKUSH. The study population was all sampled essential hypertensive patients who were attending follow-up at the outpatient department and fulfilled the inclusion criteria. Essential hypertensive patients with pregnancy, those who are critically ill and patients who had been diagnosed with renal disease, malignancy and were on chemotherapy were excluded from the study.

### Data collection procedures

Interviewer-administered structured questionnaires were used to collect socio-demographic, clinical and behaviour-related factors, as well as anthropometric data. Two BSc nurses with previous experience of data collection were recruited for data collection. Two days training was given to the data collectors prior to the data collection period to familiar them with the objective of the study. Continuous follow-up and supervision were provided by the supervisor and principal investigator throughout the data collection periods.

### Sample collection and processing

After obtaining written informed consent from the study participant in the study, the vein in which we took blood was disinfected with 70% alcohol and a tourniquet was applied. Then, 5 mL of fasting blood was collected from each study participant by a trained health professional using sterile technique. After the required amount of blood had been collected, the tourniquet was released. The drawn sample was left for 30 minutes, and then serum was separated from the collected blood sample by centrifugation at 3000 rpm for 10 minutes using a Rotanta 960 centrifuge in a thermo-stable condition. Then, serum was taken and stored at −20°C in the central Laboratory till the time of biochemical analysis.

### Laboratory test principles

Serum uric acid, glucose and lipid profile content were determined using standard principles and procedures with an ABX Pentra 400 automated chemistry analyser according to the reagent manufacturer’s instruction in Wolkite University specialized hospital. Serum uric acid is oxidized to allantoin and hydrogen peroxide by the enzyme uricase. In the presence of peroxidase, released hydrogen peroxide is coupled with aniline derivative and 4-amino antipyrine to form a colored chromogenic complex. The absorbance of the colored dye was measured at 520 nm and is proportional to the concentration of SUA in the sample.

### Anthropometry and blood pressure measurements

Body weight and height were measured according to the WHO guideline manual. Body weight was measured to the nearest 0.1kg using portable weighing scales. Subjects were weighed barefoot and wearing very light indoor clothing. Standing height without shoes was measured by an adjustable wooden measuring board. Body mass index (BMI) was calculated as the weight in kilograms (kg) divided by the square of the height in meters (m^2^). Waist circumference (WC) was measured at the midpoint between the lower margin of the least palpable rib and the top of the hip or minimal waist using stretch-resistant tape. The cut-off point for waist circumference ≥ 102cm for men and ≥ 88 cm for women were used to indicate central obesity [24]. Hip circumference was measured at the widest part of the buttocks. Blood pressure was taken using a mercury sphygmomanometer from the left upper arm after the participant was seated quietly for 5 min using a mercury-based sphygmomanometer after the subjects had rested for more than 10 minutes. For those study participants with systolic blood pressure (SBP) ≥140 mmHg and diastolic blood pressure (DBP) ≥90 mmHg, the blood pressure was measured again and the average value was taken [1].

### Operational definitions

Hypertension; was defined as SBP ≥140 mmHg or DBP ≥90 mmHg [1]. Stage 1 hypertensive patient includes patients with systolic blood pressure 140–159 mmHg or diastolic blood pressure 90–99 mmHg and Stage 2 includes patients with systolic blood pressure ≥160 mmHg or diastolic blood pressure ≥100 mmHg [25].

Hyperuricemia; was defined as a serum UA level >7.2 mg/dL for males and >6 mg/dL for females and participants with serum UA level <3.5mg/dl for males and<2.2mg/dl for females considered as hypouricemia [7]. Physical inactivity implies subjects who were not participated in moderate daily physical exercise or activities such as walking, cycling, or doing that had important benefits for health with expenditure of energy or those participants who were not involved recommended 30–60 minutes aerobic exercise 3–4 times per week to promote cardiovascular fitness [26].

Smoker represent a subject who had smoking history with one or more manufactured or hand rolled tobacco during data collection. Alcohol users refers a participant who drinks more than three to four units for males and more than two to three units for females daily which results in thinking abnormality [24].

### Data quality management

All investigators supervised data and specimen collectors, directly involve, and control any kind of procedures and processes that may affect the result. The specimen was collected, stored and transported according to the guideline and the suspected specimen in terms of poor quality was rejected automatically. Working and acceptable commercial kits were used. The daily performances were reported to supervisors and checked and cross checked timely. Measuring instruments and biochemical analyzers were calibrated by their respective reference materials. Two days training on the contents of the questionnaire, data collection techniques, and research ethics was given for those assistants and any doubts/question in the method they were undertook and clarified. Pretest of the questionnaire was conducted in 5% study subjects at Atat Hospital; Gurage Zone for validation of questionnaire two weeks prior to actual data collection and some adjustment on additional preparations was done. After checking the expiry date of both the reagents and controls, ABX Pentra 400 clinical chemistry analyzer was checked for delivering correct result by using normal and pathological controls. Before any patient sample processed, dual quality controls (normal and pathological) was performed and the patient result was taken after the controls passed. All necessary procedures and steps were followed based on the manufacture instructions. Collected results were checked for completeness on daily basis by the principal investigator.

### Statistical analysis

After that, data were coded, cleaned and entered into EpiData version 3.1 and then exported to IBM SPSS version 21 for analysis. Frequencies tables, descriptive statics were computed to describe the relevant variables. A binary logistic regression model was computed to determine the association between independent variables and hyperuricemia. Bivariable analysis was carried out for each independent variable and those variables with p-values <0.25 were identified as candidates for multivariable logistic regression model. Finally, multivariable logistic regression analysis was performed to control confounding variables and identifies significantly associated variables with hyperuricemia. Unadjusted odds and adjusted odds ratio (AOR) were computed. The 95% CI was used and a p-value of <0.05 was considered significant in multivariate logistic regression. Appropriateness of model fitness was also checked using the Hosmer–Lemeshow test.

## Results

### Distribution of socio-demographic and clinical characteristics

Among a total of 270 essential hypertensive patients, 48.5% (n = 131) females, and 51.5% (139) males were enrolled in the current study; their mean age was 46.9 ± 14.8 years. The majority of the study participants 51.5% (139) were in ≥50years of age groups. About 51.5% (139), 82.2% (222), and 50% (135) participants were urban dwellers, married, and with no formal education, respectively. When we come to BMI level, about half (44.1% (119)) of participants were overweight and 138(51.1%) were centrally obese. The mean ±standard deviation (SD) of the central obesity and BMI was 98.26±(8.22) and 26.2±(3.22) respectively. Regarding the clinical characteristics if study participants; 117(43.3%), 156(57.8%) hyperuricemic patients were hypertriglycemic and hyperglycaemic respectively.

Concerning the overall magnitude of hyperuricemia disorders among essential hypertensive patients in outpatient department was 196(27.4%) and 3(1.1%) was hypouricemic. The average serum uric acid level of participants was 6.11mg/dl (±1.05). The prevalence of hyperuricemia was highest among age groups ≥50 years 57(41%) and males 47(33.8%). The proportion of hyperuricemia was 28.6%, 57.6%, 29.3%in cigarette smokers, alcohol drinkers and chat chewers respectively. Furthermore those participants with higher BMI and centrally obese had more hyperuricemia (**Table 1**).

**Table 1:** Distribution of socio-demographic and clinical characteristics of study participants.

Variables	Categories	Frequency with(%) n = 270	Hyperuricemia (%) with n = 270		p-value
			Yes = 1	No = 0	
Age	> = 50	139(51.5)	57(41)	82(59)	0.00
	25–49	87(32.2)	14(16.1)	73(83.9)	0.00
	< = 25	44(16.3)	3(6.8)	41(93.2)	0.001
Sex	Male	139(51.5)	47(33.8)	92(66.2)	0.015
	Female	131(48.5)	27(20.6)	104(79.4)	0.022
Residence	Urban	139(51.5)	37(27.7)	102(72.3)	0.923
	Rural	131(48.5)	35(27.1)	94(72.9)	0.987
Occupational status	Farmer	49(18.1)	10(20.4)	39(79.6)	0.485
	Daily labourer	18(6.7)	7(38.9)	11(61.1)	0.486
	Merchant	132(48.9)	37(28)	95(72)	0.176
	civil servant	71(26.3)	20(28.2)	51(71.8)	0.832
Educational status	No formal education	135(50)	35(25.9)	100(74.1)	0.391
	Primary	56(20.7)	19(33.9)	37(66.1)	0.388
	Secondary	45(16.7)	9(20)	36(80)	0.498
	Higher	34(12.6)	11(32.4)	23(67.6)	0.798
Marital status	Married	222(82.2)	54(24.3)	168(75.7)	0.611
	Single	26(9.6)	9(34.6)	17(65.4)	0.802
	Widowed	14(5.2)	7(50)	7(50)	0.196
	Divorced	8(3)	4(50)	4(50)	0.201
Cigarette smoking	Yes	28(10.4)	8(28.6)	20(71.4)	0.884
	No	242(89.6)	66(27.3)	176(72.7)	0.901
Alcohol consumption	Yes	92(34)	53(57.6)	39(42.4)	0.000
	No	178(66)	21(11.8)	157(88.2)	0.000
Khat chewing	Yes	82(30.4)	24(29.3)	58(70.7)	0.652
	No	188(69.6)	50(26.6)	138(73.4)	0.761
Physical activity	No	158(58.5)	38(24.1)	120(75.9)	0.143
	Yes	112(41.5)	36(32.1)	76(67.9)	0.761
Medication	Yes	148(54.8)	63(42.6)	85(57.4)	0.000
	No	122(45.2)	11(9)	111(91)	0.000
Body mass	> = 30	41(15.2)	28(68.3)	13(31.7)	0.000
index status (Kg/m^2^)	25–29.9	119(44.1)	33(27.7)	86(72.3)	0.023
	18.5–24.9	83(30.7)	8(9.6)	75(90.4)	0.012
	<18.5	27(10)	5(18.5)	22(81.5)	0.011
Central obesity	Yes	138(51.1)	63(45.7)	75(54.3)	0.000
	No	132(48.9)	11(8.3)	121(91.7)	0.000
Stages of hypertension	Stage-2	152(56.3)	53(34.9)	99(65.1)	0.002
	Stage-1	118(43.7)	21(17.8)	97(82.2)	0.003

### Correlation analysis between hyperuricemia and clinical predictor variables

The correlation analysis between serum uric acid concentration and independent clinical predictors was assessed. Based on the assessment, level of serum uric acid showed strong positive correlation with fasting blood sugar and triglyceride levels(r = 0.302, p = 0.000) and(r = 0.332, p = 0.000) respectively. Low density lipoprotein cholesterol (LDL-c) showed significant strong positive correlation with serum uric acid level (r = -0.46, P = 0.000) (**Table 2**).

**Table 2:** Correlation analysis between serum uric acid and clinical predictors.

Clinical predictor variables	Mean±(SD)	Uric acid levels	
		R	p-value
TG	140±(27.02)	0.332	0.000
LDL-C	100.28±(10.23)	0.461	0.000
HDL-C	41.86±(8.61)	0.083	0.175
FBS	118.032±(2.01)	0.302	0.000

r = Pearson’s correlation coefficient, p = p-value for correlation, TG, triglyceride; HDL-C, high density lipoprotein; LDL-C, low density lipoprotein; FBS, fasting blood sugar.

### Factors associated with hyperuricemia in bivariable logistic regression

In bivariable analysis model by considering p-value<0.25, higher age (COR (95% CI) = 9.5(2.8,22.02), being a male (COR (95% CI) = 1.96(1.13,3.41), alcohol consumers (COR (95% CI) = 10.16(5.49,18.79), study subjects with drugs (COR (95% CI) = 7.47(3.71,15.06), higher BMI levels (COR (95% CI) = 9.47(2.93,18.62, being centrally obese (COR (95% CI) = 9.24(4.57,18.64) and higher stages of hypertension (COR (95% CI) = 2.47(1.38,4.40) were identified as candidate variables to be tested for association with in multivariable logistic analysis **(Table 3).**

**Table 3:** Bivariate analysis of factors associated with hyperuricemia among essential hypertensive patients attending their follow-up at the outpatient department, in Wolkite University specialized hospital, Southern Ethiopia from November 1 to February 30, 2021.

Variables	Categories	Hyperuricemia (%)		p-value	Unadjusted OR (95% CI)
		Yes = 1	No = 0		
Age	> = 50	57(41)	82(59)	0.00	9.5(2.8,22.02)*
	25–49	14(16.1)	73(83.9)	0.148	2.62(0.71,9.65)
	< = 25	3(6.8)	41(93.2)	1	
Sex	Male	47(33.8)	92(66.2)	0.016	1.96(1.13,3.41)*
	Female	27(20.6)	104(79.4)	1	1
Residence	Urban	37(27.7)	102(72.3)	0.92	1.02(0.60,1.75)
	Rural	35(27.1)	94(72.9)	1	1
Occupational status	Farmer	10(20.4)	39(79.6)	0.120	0.40(0.12,1.30)
	Daily labourer	7(38.9)	11(61.1)	0.341	0.61(0.22,1.69)
	Merchant	37(28)	95(72)	0.372	0.37(0.20,1.81)
	civil servant	20(28.2)	51(71.8)		1
Educational status	No formal education	35(25.9)	100(74.1)	0.45	0.73(0.32,1.65)
	Primary	19(33.9)	37(66.1)	0.87	1.07(0.43,2.65)
	Secondary	9(20)	36(80)	0.21	0.52(0.18,1.45)
	Higher	11(32.4)	23(67.6)	1	1
Marital status	Married	54(24.3)	168(75.7)	0.117	1.12(0.21,5.97)
	Single	9(34.6)	17(65.4)	0.437	0.44(0.10,1.88)
	Widowed	7(50)	7(50)	1.000	0.24(0.04,0.51)
	Divorced	4(50)	4(50)	1	1
Cigarette smoking	Yes	8(28.6)	20(71.4)	0.884	1.06(0.44, 2.53)
	No	66(27.3)	176(72.7)	1	1
Alcohol consumption	Yes	53(57.6)	39(42.4)	0.000	10.16(5.49,18.79)*
	No	21(11.8)	157(88.2)	1	1
Khat chewing	Yes	24(29.3)	58(70.7)	0.394	1.14(0.64,2.03)
	No	50(26.6)	138(73.4)	1	1
Physical activity	No	38(24.1)	120(75.9)	0.143	0.66(0.39,1.14)
	Yes	36(32.1)	76(67.9)	1	1
Medication	Yes	63(42.6)	85(57.4)	0.000	7.47(3.71,15.06)*
	No	11(9)	111(91)	1	1
Body mass index status (Kg/m^2^)	> = 30	28(68.3)	13(31.7)	0.000	9.47(2.93,18.62)*
	25–29.9	33(27.7)	86(72.3)	0.329	1.68(0.59,4.82)
	18.5–24.9	8(9.6)	75(90.4)	0.222	0.46(0.139,1.58)
	<18.5	5(18.5)	22(81.5)	1	1
Central obesity	Yes	63(45.7)	75(54.3)	0.000	9.24(4.57,18.64)*
	No	11(8.3)	121(91.7)	1	1
Stages of hypertension	Stage-2	53(34.9)	99(65.1)	0.002	2.47(1.38,4.40)*
	Stage-1	21(17.8)	97(82.2)	1	1

* = Statistically significant variable at p<0.25, 1 = Reference category, COR = Crud Odds Ratio.

### Predictors significantly associated with hyperuricemia in multivariable logistic regression analysis

Multivariate logistic regression models were employed to identify the independent predictors of hyperuricemia in essential hypertensive patients. After adjusting for other predictor variables: alcoholic patients were ~ nearly 16 times more likely to develop hyperuricemia (AOR: 15.68, 95% CI: (5.93, 21.41) than non-alcoholic groups. Hypertensive patients who took antihypertensive drugs were higher odds of having hyperuricemia (AOR: 11.56, 95% CI: (3.94, 23.80) than hypertensive patients without medication. Patients with higher BMI were more likely to develop hyperuricemia (AOR: 4.89, 95% CI: (1.46, 25.5) compared to their counterparts and those centrally obese hypertensive patients were higher odds of having hyperuricemia (AOR: 6.87, 95% CI: (2.53, 18.63) compared to non-centrally obese hypertensive patients **(Table 4).**

**Table 4:** Multivariate analysis of factors associated with hyperuricemia among adult essential hypertensive patients at Wolkite University specialized hospital, Wolkite, Southern Ethiopia, from November 1 to February 30, 2021.

Variables	Categories	Hyperuricemia (%)		p-value	AOR (95% CI)
		Yes = 1	No = 0		
Age	> = 50	57(41)	82(59)	0.109	3.49(0.75,16.17)
	25–49	14(16.1)	73(83.9)	0.479	0.54(0.101,2.93)
	< = 25	3(6.8)	41(93.2)	1	
Sex	Male	47(33.8)	92(66.2)	0.933	1.03(0.44,2.44)
	Female	27(20.6)	104(79.4)	1	1
Alcohol consumption	Yes	53(57.6)	39(42.4)	0.000	15.68(5.93,21.41)**
	No	21(11.8)	157(88.2)	1	1
Medication	Yes	63(42.6)	85(57.4)	0.000	11.56(3.94,23.80)**
	No	11(9)	111(91)	1	1
Body mass index status (Kg/m^2^)	> = 30	28(68.3)	13(31.7)	0.048	4.89(1.46,25.5)**
	25–29.9	33(27.7)	86(72.3)	0.455	1.75(0.40,7.68)
	18.5–24.9	8(9.6)	75(90.4)	0.243	0.36(0.0.06,1.97)
	<18.5	5(18.5)	22(81.5)	1	1
Central obesity	Yes	63(45.7)	75(54.3)	0.000	6.87(2.53,18.63)**
	No	11(8.3)	121(91.7)	1	1
Stages of hypertension	Stage-2	53(34.9)	99(65.1)	0.374	1.52(0.60,3.84)
	Stage-1	21(17.8)	97(82.2)	1	1

** = statistically significant at p <0.05, 1 = Reference category, AOR = Adjusted Odds Ratio.

## Discussion

Hypertension and other cardiovascular complications have played great attention as a potential clinical condition predicting the development of hyperuricemia in metabolic syndrome patients and in turn compromising the management of the disease itself and its progression [5]. In previous studies, moderately raised levels of serum uric acid have been taken as a simple biochemical problem in essential hypertensive patients with little clinical significance. However, recently, it has become increasingly clear that moderately elevated levels of serum uric acid are significantly associated with increased cardiovascular morbidity and mortality by causing stressful conditions and different debilitating complications in essential hypertensive patients [19]. The main finding of this study was high prevalence of SUA concentration among essential hypertensive patients and significant association between high serum uric acid levels and various types and increased frequency of factors in study participants.

In this study, the magnitude of SUA among essential hypertensive patients was 27.4%. The findings of this study was lower as compared to studies conducted in University of Gondar Hospital, Northwest Ethiopia, Hawassa university comprehensive specialized hospital, South West Ethiopia and Guangdong Province in China where 31.5%, 33.8%, 32.6 of study participants were found to be hyperuricemic respectively [10,12,23]. Also a similar study was done Nobel Medical College, Biratnagar district of Nepal where the magnitude of hyperuricemia was found to be 28.57% [18]. The variation attributed might be different socio-demographic and behavioural characteristics, and the inclusion of patients with other metabolic syndromes with their complications and in some of the studies inclusion of participants admitted with inpatient therapies that ameliorate hyperuricemia.

However, the result was higher when compared to the study done in Type 2 Diabetes Mellitus Patients Attending Jimma Medical Center, South-western Ethiopia and in urban area of Beijing, China where the prevalence of hyperuricemia was found to be 16.7% and 22% respectively [13,20]. The variation might be attributed to the majority of participants in this were diabetics with complication in which, patients who have insulin resistance with decrease in excretion of UA due to the reduced effects of insulin action [27]. In addition variability of the study subjects may contribute for magnitude difference.

This study revealed that study participants with alcoholic were found to increase the odds of hyperuricemia by nearly 16 folds (AOR: 15.68, 95% CI: (5.93, 21.41)) than non-alcoholics. This was in line with studies conducted south west Ethiopia and Seychelles. The possible justification could be, as participants got more alcoholic there is more production of SUA concentrations by increased generation of NADH as a result of oxidation of ethanol to acetaldehyde which in turn enhance the conversion of pyruvate to lactate that causes more urate production. Being alcoholic also reduces excretion as an immediate action [19,20,28].

Also, subjects who took anti-hypertensive medication have significantly associated with hyperuricemia in which the likely hood of developing hyperuricemia were 11.56 higher in participants with medication when compared with counterparts. This was in line with studies conducted in Tunisia, Seychelles and Pakistan [19,29,30]. The association might be dues to the fact that medications especially diuretics, angiotensin converting enzyme inhibitors and beta-blockers are most frequently prescribed and used antihypertensive drug in outpatient department to reduce complications secondary to essential hypertension. However, despite achieving adequate control a rise in blood pressure, these drugs are also associated with adverse events like hyperuricemia. Diuretics, beta-blockers and angiotensin converting enzyme inhibitors are associated with elevated serum uric acid by increasing direct urate reabsorption in the proximal renal tubules and by inhibited basolateral organic anion transporters and by reducing glomerular filtration rate [5,29].

The current study also showed that higher BMI and being centrally obese had significantly associated with hyperuricemia (AOR: 11.56, 95%CI: (3.94, 23.80)) and (AOR: 6.87, 95%CI: (2.53, 18.63)) respectively. This was supported with similar studies conducted in Ethiopia, Japan and Bangladesh [7,16]. The possible explanation could be higher BMI and being centrally obese or excessive body fat may be related to excessive uric acid production and its poor excretion, due to insulin resistance, resulting in impaired uric acid metabolism to the level of hyperuricemia. Those patients with insulin resistance secrete larger amounts of insulin to maintain an adequate glucose metabolism in negative feedback mechanism and the kidney responds to the high insulin levels by decreasing uric acid clearance, probably linked to insulin-induced urinary sodium retention in centrally obese patients. Meanwhile, hyperuricemia in turn can induce obesity by enhancing liver and peripheral fat production [22,27,31]. Although, the necessary opportunities were made to decrease the possible limitations of this study, the interpretation of result should be in light of the following limitations. Data on pattern of diet was assessed. There might be recall bias since study subjects were asked for situations happened in the past. Causality links was prohibited due to cross sectional nature of the study. Furthermore, this study might be among a few which tried to evaluate hyperuricemia in essential hypertensive patients.

## Conclusion

In this study, the high burden of hyperuricemia (27.4%) was observed in essential hypertensive patients with follow-up in outpatient department. Taking alcohol and antihypertensive medication, being overweight and centrally obese were identified factors of hyperuricemia. The finding of this study should be taken into consideration to implement preventive interventions on identified predictors in hypertensive patients. Taking fruit and vegetable, and promoting physical exercise and determinations of serum uric acid level in adult essential hypertensive patients was recommended to minimize the emergence of hyperuricemia. The most important management of these derangements can best be achieved by averting the underlying pathophysiologic events.

## Ethical consideration

Ethical clearance was obtained from Wolkite University intuitional review board; concerned administrative offices were communicated with written formal letter. Written informed consent was obtained from each study subjects after explaining the purpose and procedures of the study before enrolment, and those willing to participate were inclouded. Confidentiality of the information was assured and the privacy of the study groups was assured by keeping their response anonymous. Based on laboratory results, study subjects with the severe forms of uric acid disorder were referred to physicians for further care.

## Supporting information

S1 File (SAV)
